# APEX: an Annotation Propagation Workflow through Multiple Experimental Networks to Improve the Annotation of New Metabolite Classes in *Caenorhabditis elegans*

**DOI:** 10.1021/acs.analchem.3c02797

**Published:** 2023-11-20

**Authors:** Liesa Salzer, Elva María Novoa-del-Toro, Clément Frainay, Kohar Annie B Kissoyan, Fabien Jourdan, Katja Dierking, Michael Witting

**Affiliations:** † Research Unit Analytical BioGeoChemistry, Helmholtz Zentrum München, 85764 Neuherberg, Germany; ‡ Toxalim (Research Centre in Food Toxicology), Université de Toulouse, INRAE, ENVT, INP-Purpan, UPS, 180 chemin de Tournefeuille St-Martin-du-Touch, BP 3, 31931 Toulouse Cedex, France; § Department of Evolutionary Ecology and Genetics, Zoological Institute, Kiel University, 24118 Kiel, Germany; ∥ MetaToul-MetaboHUB, National Infrastructure of Metabolomics and Fluxomics, 180 chemin de Tournefeuille St-Martin-du-Touch, BP 3, 31931 Toulouse Cedex, France; ⊥ Metabolomics and Proteomics Core, Helmholtz Zentrum München, 85764 Neuherberg, Germany; # Chair of Analytical Food Chemistry, TUM School of Life Sciences, Technical University of Munich, 85354 Freising-Weihenstephan, Germany

## Abstract

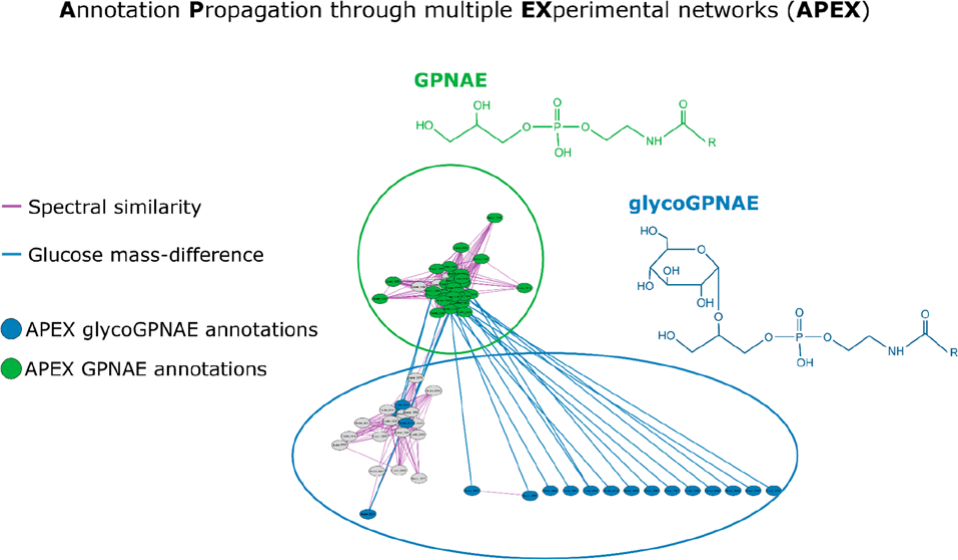

Spectral similarity networks, also known as molecular networks, are crucial in non-targeted metabolomics to aid identification of unknowns aiming to establish a potential structural relation between different metabolite features. However, too extensive differences in compound structures can lead to separate clusters, complicating annotation. To address this challenge, we developed an automated Annotation Propagation through multiple EXperimental Networks (APEX) workflow, which integrates spectral similarity networks with mass difference networks and homologous series. The incorporation of multiple network tools improved annotation quality, as evidenced by high matching rates of the molecular formula derived by SIRIUS. The selection of manual annotations as the Seed Nodes Set (SNS) significantly influenced APEX annotations, with a higher number of seed nodes enhancing the annotation process. We applied APEX to different *Caenorhabditis elegans* metabolomics data sets as a proof-of-principle for the effective and comprehensive annotation of glycerophospho *N*-acyl ethanolamides (GPNAEs) and their glyco-variants. Furthermore, we demonstrated the workflow’s applicability to two other, well-described metabolite classes in *C. elegans*, specifically ascarosides and modular glycosides (MOGLs), using an additional publicly available data set. In summary, the APEX workflow presents a powerful approach for metabolite annotation and identification by leveraging multiple experimental networks. By refining the SNS selection and integrating diverse networks, APEX holds promise for comprehensive annotation in metabolomics research, enabling a deeper understanding of the metabolome.

## Introduction

Networks have emerged as a powerful formalism for modeling and analyzing complex systems of interacting elements. A network is a collection of nodes connected by edges that represent interactions or relationships between them. When a particular phenomenon (such as metabolism) can be modeled as a network, the topology of such a network can be used to study the phenomenon. There are different ways to build metabolism-related networks, but they can be broadly divided into knowledge-based and experimental networks.^1^

Knowledge-based networks, such as the Genome-Scale Metabolic Network (GSMN), aggregate knowledge about the metabolism of a specific organism, e.g., human or *Caenorhabditis elegans*.^2−4^ The GSMN is constructed on the basis of annotated genomes.

In contrast, experimental networks can be generated from metabolomics data, i.e., holistic measurements that systematically measure and (semi)quantify all metabolites present in a given sample, aiming to correlate changes in metabolite intensities or concentrations with physiological phenotypes. Known and unknown metabolic reactions and pathways were reconstructed from metabolomics data. Here, we are focusing on liquid chromatography–tandem mass spectrometry (LC-MS/MS)-based nontargeted metabolomics, with which different experimental networks can be (re)constructed, such as mass difference and spectral similarity networks; all of which can be used to interpret the obtained metabolomics data.

Mass difference networks use exact *m*/*z* values and the corresponding pairwise mass differences (represented as edges).^5−7^ These mass differences are compared against a list of known mass differences corresponding to the biochemical transformations of interest. For instance, a mass difference of 15.9949 could indicate the gain or loss of an oxygen atom, and if such a biochemical transformation is of interest, a connection between the corresponding nodes is added. However, although the mass difference between a pair of metabolite features could be explained by the biochemical transformation link between them, this is not necessarily the case. Two metabolite features may have the same mass difference as a biochemical transformation of interest only by chance or because of unrelated biochemical transformations. Different techniques could be used to improve the quality of a mass difference network (i.e., to reduce the false positive edges), for instance, homologous series. Homologous series are a group of compounds that differ from each other by a specific repeating unit, such as a CH_2_ group in a homologous series of fatty acids.^8^ Retention time is used to identify those homologous series based on a consistent trend observed in liquid chromatography (LC) separations, for example, in reversed-phase separations, wherein the retention time tends to increase as the number of CH_2_ units increase. If unknown metabolites are connected in the mass difference network and are part of a homologous series, it provides strong evidence for the identities of the unknown metabolites. It is to note that, although mass difference networks can be a useful tool for metabolite annotation and identification, they do not consider any structural relation between metabolites.

In contrast, spectral similarity networks, also known as molecular networks, are based on spectral patterns from fragmentation experiments that can incorporate some aspects of chemical structural information and can provide more accurate metabolite annotation. The nodes represent corresponding MS^2^ spectra of metabolite features, which are compared by their spectral similarity, with different scoring metrics available.^9,10^ An edge between two nodes is drawn if the spectral similarity between the corresponding fragmentation spectra is above a specific threshold. It is important to note that the topology of a spectral similarity network is dependent on the metric used for comparing the MS^2^ spectra and the threshold. Thus, if spectra are too dissimilar, no connection can be added, even if potential biochemical connections exist.

Correct annotation and identification of metabolites in nontargeted metabolomics remains as one of the primary challenges in the field, and different types of networks, covering different aspects of the biology, serve as valuable tools to aid in this task, especially when combined.

In the present work, we introduce an automated Annotation Propagation through a multiple EXperimental networks (APEX) workflow. Our workflow combines spectral similarity networks with mass difference networks and application of homologous series. The aim of APEX is to bridge between different types of networks and to allow propagating the annotations beyond a single network to uncover new potential biological links useful for metabolite annotation and identification as well as biological interpretation.

In this study, we utilize the APEX workflow to aid in the identification of glycerophospho *N*-acyl ethanolamides (GPNAEs), a recently discovered compound class in *Caenorhabditis elegans* (*C. elegans*), that has been identified in starved larvae and peroxisomal α-oxidation mutants.^11,12^ GPNAEs are intermediates in the synthesis of *N*-acyl ethanolamines (NAEs), which are linked to lifespan extension in the nematode.^13^ Due to their recent discovery, no deposited reference spectra and no chemical reference standards for GPNAEs are yet commercially available. Notably, a characteristic fragmentation pattern of GPNAEs and its acyl chain variants connect the corresponding nodes in spectral similarity networks as performed by Helf et al. However, differences in their structure upon specific biochemical transformation (glycosylation) changes abundance of common fragments and introduces new fragment peaks as well, which results in separated clusters in molecular networks, limiting the ability to propagate annotations between them.^12^ Nevertheless, the combination of different experimental networks allows one to bridge between such seemingly unrelated clusters.

Using the APEX workflow, we improved species identification within the GPNAE compound class in different *C. elegans* data sets. We also evaluated its effectiveness for annotating species from two other *C. elegans* metabolite classes, namely, ascarosides and modular glycosides (MOGLs). Our results highlight the potential of APEX for annotating GPNAEs and its implications for future metabolomics research.

## Material and Methods

### Chemicals

Methanol (MeOH), isopropanol (iPrOH), acetonitrile (ACN), and formic acid have been of LC-MS grade and purchased from Sigma-Aldrich (Sigma-Aldrich, Taufkirchen, Germany). Water was purified from a Millipore Integral 3 water purification system with a TOC < 3 ppb and >18.2 MOhm.

### *C. elegans* Culture

The *C. elegans* N2 strain was maintained on nematode growth medium at 20 °C according to the routine protocol.^14^*Pseudomonas lurida* MYb11, *Pseudomonas fluorescens* MYb115, and *Escherichia coli* OP50 were grown on Tryptic Soy Agar (TSA) at 25 °C. Worms were grown on 9 cm Peptone Free Nematode Growth Medium (PFM) plates with a bacterial lawn (OD_600_ = 10) of either MYb11, Myb115, or OP50 at 20 °C for at least two generations. Four biological replicates were used for each treatment group. Each replicate consisted of 1000 to 1500 synchronized hermaphrodites at the first larval stage (L1) pipetted onto the bacterial lawns. Two days later, the worms were transferred to plates containing OP50. Worms were harvested after 24 h by thoroughly washing each plate with chilled M9, followed by centrifugation at 3500 rpm for 1 min. The pellet was collected and washed four more times. Finally, the pellets were transferred into 1 mL of H_2_O/MeOH (50/50, v/v) and flash-frozen in liquid N_2_.

### Metabolite Extraction

After worm samples had been thawed on ice, they were transferred to bead beating tubes and homogenized using a Precellys beat beating system with a Cryolys cooling module (Bertin Technologies). After homogenization, samples were centrifuged for 15 min at 15000 rpm at 4 °C. The supernatant was transferred to a fresh reaction tube and evaporated to dryness using a Speedvac (Thermo Savant). Samples were stored dry at −80 °C until analysis. From the residue, protein quantities were determined using a bicinchoninic acid (BCA) kit (Sigma). Prior to analysis, samples were redissolved in 50 μL of 80% H_2_O/20% ACN. A total of 40 μL was transferred to an autosampler vial, and 10 μL from each sample was mixed for a pooled quality control (QC) sample.

### UPLC-UHR-TOF-MS Analysis of *C. elegans* Microbiota Samples

Metabolite extracts were analyzed on a Waters Acquity UPLC (Waters, Eschborn, Germany) coupled to a Bruker maxis UHR-TOF-MS instrument (Bruker Daltonics, Bremen, Germany). Separation was achieved on a Waters Acquity BEH C18 column (100 mm × 2.1 mm ID, 1.7 μm particle size). Eluent A consisted of 100% H_2_O/0.1% formic acid and Eluent B of 100% ACN/0.1% formic acid. Gradient conditions were as follows: 95/5 at 0.0 min, 95/5 at 1.12 min, 0.5/99.5 at 6.41 min, 0.5/99.5 at 10.01 min, 95/5 at 10.1 min, and 95/5 at 15.0 min. Detection was carried out in positive and negative ionization modes using data-dependent acquisition. MS parameters were as follows: End-plate offset = −500 V, Capillary = −4500 V (positive mode)/4000 V (negative mode), Nebulizer pressure = 2.0 bar, Dry gas = 8.0 mL/min, Dry temperature = 200 °C. MS^2^ spectra were acquired with data-dependent acquisition using Bruker AutoMSn with default parameters for the isolation window and collision energy ramping. For individual recalibration of each chromatogram, 1:4 diluted low concentration tune mix (Agilent, Waldbronn, Germany) was injected via a six-port valve before each run between 0.1 and 0.3 min.

### Data Preprocessing

All data sets (*C. elegans* microbiota, MSV000087885 and MSV000086293) were processed the same way using Genedata Expressionist for MSMS 13.5.4 (Genedata AG, Basel, Switzerland). Processing included chemical noise subtraction, retention time alignment, isotope clustering, peak detection, and grouping. The resulting feature table and corresponding MS^2^ spectra were exported and used to build the experimental networks and to manually annotate the metabolite features that were used as seeds for the APEX workflow (i.e, the GPNAE, the ascaroside, and MOGL compounds), as described in the following sections.

### Construction of Mass Difference Networks, Homologous Series, And Spectral Similarity Networks

Mass difference networks were created using the *MetNet* R package^15^ and upon mass matching of 10 and 5 ppm tolerance for the qToF and Orbitrap data, respectively, using a list of 27 mass difference of biotransformations that might be a relevant GPNAE metabolism (Table S1) and 21 mass differences relevant to ascaroside/MOGL metabolism (Table S2).

Homologous series have been calculated using the *nontarget* R package (https://github.com/blosloos/nontarget), considering only C, H, and O for the mass difference. Even more, the minimum *m*/*z* difference was 5 Da, the maximum *m*/*z* difference was 60 Da with a tolerance of 5 Da, the minimum RT shift was 12 s, the maximum was 60 s with a tolerance of 5 s, and there was a minimum of 4 features per homologous series cluster.

The spectral similarity networks were generated using Feature Based Molecular Networking (FBMN) in GNPS.^16^ The feature table and MS^2^ spectra were formatted to be compatible with XCMS input format for FBMN. Settings were as follows: mass tolerance of 0.02 Da, minimum cosine of 0.8, maximum 1000 neighbor nodes, minimum 3 matched fragment ions, and unlimited component size.

The experimental networks spectral similarity G_s = (V_s, E_s) and mass difference G_m = (V_m, E_m) and homologous series (G_h = V_h, E_h) are then merged into a single network G_apex = (V, E), merging the duplicated vertices (of V_s + V_m + V_h) and corresponding edges E so that there is a single edge between any pair of nodes and saving the number and type of experimental networks merged as an edge attribute.

### Overview of the APEX workflow

**Figure 1:**
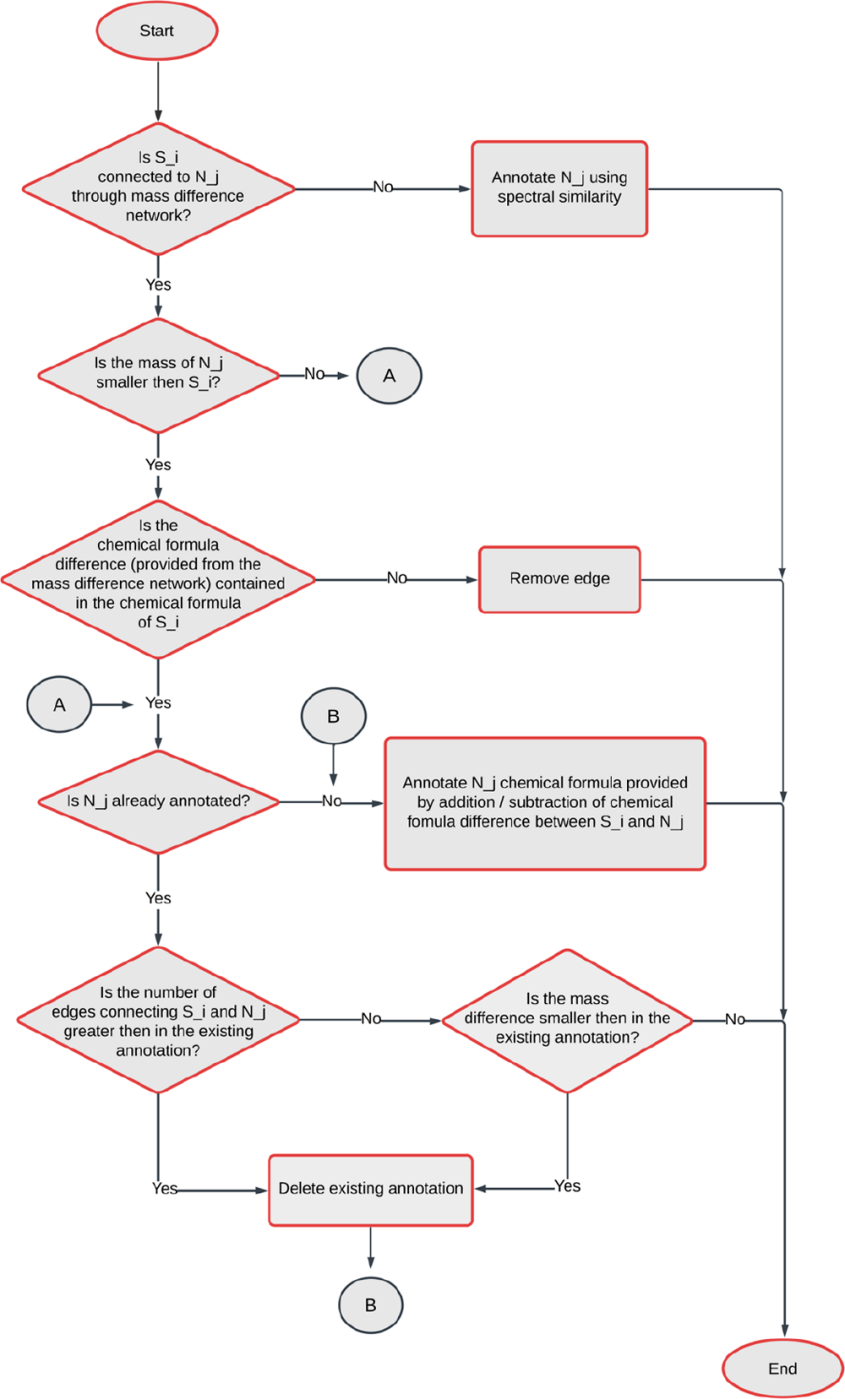
Scheme of the APEX workflow. A pseudocode explaining the algorithm can be found in the SI, S1. The workflow starts by iteration through the manually annotated seed nodes S_i to get their set of neighbors N(S_i). Then the iteration continues through all the neighbors N_j, where the scheme starts. If a neighbor N_j is annotated, new attributes are added to N_j containing the networks connecting N_j and S_i, the seed node S_i, and other available attributes (i.e., values of spectral similarity, mass difference, and homologous series).

The automated APEX workflow is schematically shown in Figure 1 and depicted as a pseudocode in SI, S1.

Overall, the APEX workflow is designed to propagate annotations from manually annotated seed nodes to their first neighbors using a combination of mass difference, spectral similarity, and homologous series. The resulting annotations are simplified (maximum of one annotation per node) to facilitate downstream analysis.

APEX is implemented in R and uses *igraph*, *Spectra*, *MsBackendMgf*, and *MetaboCoreUtils* package and is available on GitHub (https://github.com/michaelwitting/APEX) together with all relevant data from the example data sets used.

## Results and Discussion

### Data Sets

To test the effectiveness of the APEX workflow, we used three *C. elegans* metabolomics data sets. The first data set was generated in-house on a UPLC-UHR-ToF-MS using reversed phase (RPLC) separation (*C. elegans* microbiota). The second data set was taken from Helf et al.,^12^ downloaded from MassIVE (MSV000087885), and also used to annotate GPNAEs. The third and last data set was taken from Le et al.,^17^ downloaded from MassIVE (MSV000086293) and used to annotate ascarosides and modular glycosides (MOGLs) in order to test the versatility of APEX. For each data set, we performed data preprocessing, generated mass difference networks, homologous series, and spectral similarity networks, as described above. We note that APEX is agnostic of the LC-MS/MS preprocessing software and only requires a feature table and related MS^2^ spectra. Metabolites have been manually annotated by interpretation of fragmentation spectra and/or curated from the respective publications.

### Manual Annotation of GPNAEs, Ascarosides, and MOGLs

The first two data sets (*C. elegans* microbiota and MSV000087885) were screened for different GPNAE variants by exact mass matching in negative ionization mode (because of its characteristic fragmentation in negative mode), using an in-house MS^1^ library containing GPNAEs with different acyl chain lengths. In order to confirm those GPNAE variants, the corresponding MS^2^ spectra were inspected to contain several fragments: *m*/*z* 79.9668 (metaphosphoric acid, [H_2_PO_3_]^−^), *m*/*z* 171.0064 (glycerol 3-phosphate, [C_3_H_8_O_6_P]^−^), *m*/*z* 152.9958 (glycerol 3-phosphate minus water, [C_3_H_6_O_5_P]^−^), and the neutral loss of 74.0367 (−C_3_H_6_O_2_ resulting in diagnostic NAE-phosphate fragment).^18^

In total, we manually annotated 19 and 10 features as GPNAEs in the *C. elegans* microbiota and MSV000087885 data set, respectively. In the third data set, ascarosides and MOGLs have been annotated. We annotated 9 and 16 candidates (ascarosides and MOGLs, respectively) based on exact mass matching and MS^2^ fragmentation spectra. Even more we used retention time (RT) matching, if available from the respective publication yielding high confidence annotations, which can be used as seeds.^17^

### Development of the APEX Workflow

**Figure 2:**
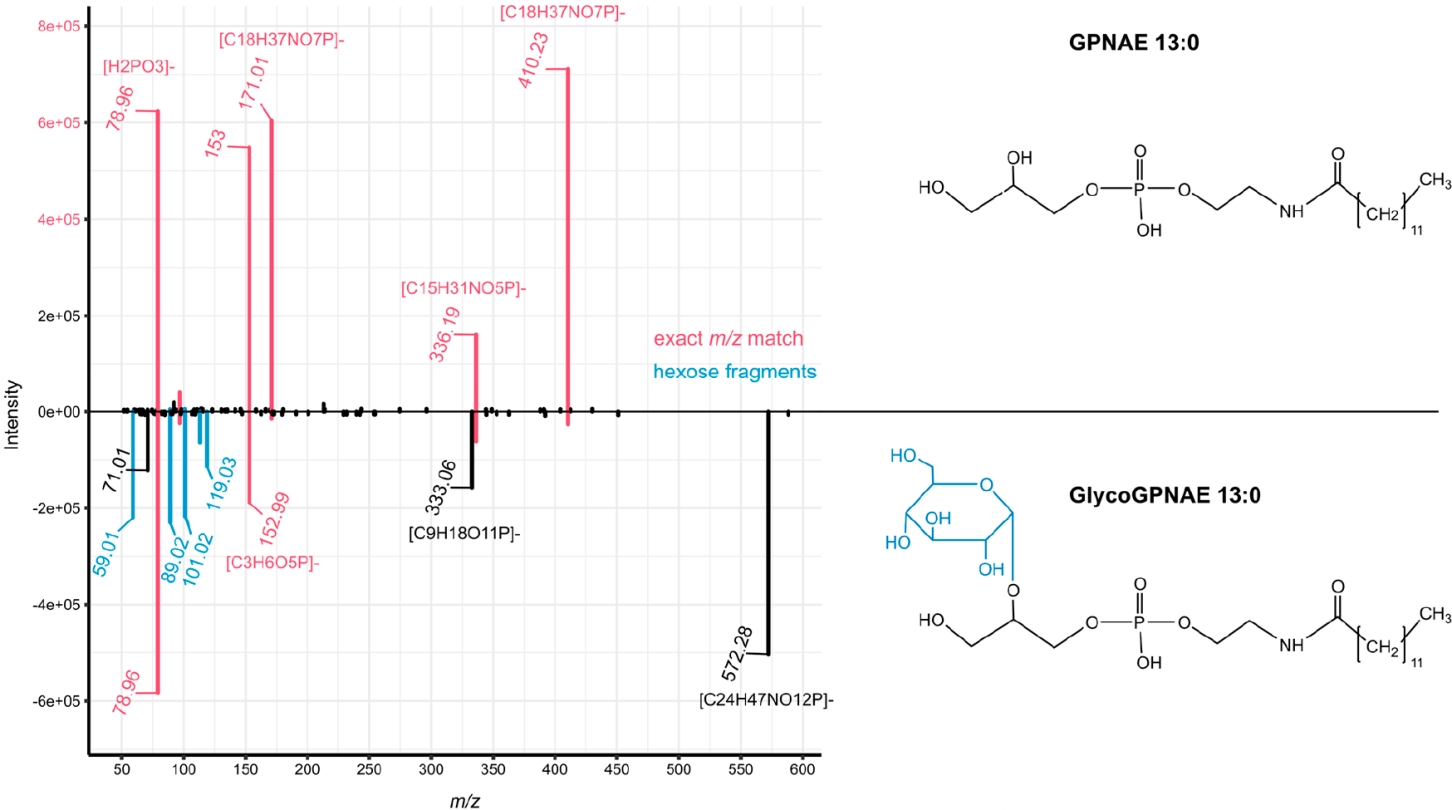
Mirror plot of GPNAE 13:0 (top spectrum) and GlycoGPNAE 13:0 (bottom spectrum) and molecular structure of (Glyco)GPNAE. Exact *m*/*z* matches are displayed in pink, and internal glucose fragments of GlycoGPNAE 13:0 are displayed in blue.

Each type of network, mass difference, or spectral similarity covers different aspects of metabolic transformations. Mass differences often can be spurious, and spectral similarity can be used to establish a potential structural similarity. However, certain structural modifications might change the fragmentation in such a way that the potential structural similarity can no longer be established. For example, comparing fragmentation of GPNAE 13:0 and GlycoGPNAE 13:0 (Figure 2), we see that those glucose variants exhibit changes in the fragmentation behavior. Even though there are several matching peaks (*m*/*z* 410.2313 [C_18_H_37_NO_7_P]^−^, 336.1940 [C_15_H_31_NO_5_P]^−^, 171.0064 [C_3_H_8_O_6_P]^−^, 152.9958 [C_3_H_6_O_5_P]^−^, and 79.9668 [H_2_PO_3_]^−^), two of which match the neutral loss of hexose (i.e., 572.2835–162.0528 = 410.2313 and 333.0592–162.0528 = 171.0064), they vary greatly in their intensity. In addition, glucose variants show additional fragments corresponding to internal glucose fragments (*m*/*z* 101.0244, 119.0708, 89.0239, and 59.0133).^19^ The resulting (modified) GNPS cosine score^9,10^ comparing the spectra of GPNAE and GlycoGPNAE is equal to 0.69 (0.64 without considering the precursor *m*/*z*), which results in the generation of separate clusters in the spectral similarity network (with a threshold for the modified cosine >0.8). Nevertheless, both features can be associated with a meaningful mass difference between the precursor *m*/*z* of 162.0528 Da corresponding to the addition of a hexose moiety.

To establish new connections between features not connected in the spectral similarity network, we used the mass difference network. However, some connections in the mass difference network could lead to incorrect conclusions; for example, connections to isomeric compounds or matches to random features that have no biological meaning. In the case of LC-MS/MS, the retention time can be used as an additional level of information.

Certain metabolite classes from homologous series, e.g., lipid-like molecules, show differences in acyl-chain length. For example, fatty acids form well-known homologous series, where each member of the series differs from the previous member by repeating the methylene (CH_2_) unit. This can be used for metabolite identification since a distinct pattern in the chromatographic separation will be found for the homologous series. In the case of reversed-phase-based separation, an increase in chain length leads to an increased retention. By grouping features that belong to the same homologous series, we can increase the reliability of some mass difference annotations linked to lipid-like compounds. Hence, we used homologous series as additional information in our APEX workflow.

In APEX, we take advantage of the different topologies of the various experimental networks to propagate and hence predict accurate annotations of specific metabolite classes such as GPNAE. Starting from a set of seed nodes, the APEX workflow iteratively annotates the first neighbors of each seed, varying the annotation based on the types of connections between the nodes.

If multiple annotations of the same node exist based on different seed nodes (i.e., manual annotations) and leading to the same predicted molecular formula, APEX prioritizes the one that considers the highest number of experimental network connections, and if equal, the simplest one with the smallest mass difference is preferred. This approach helps to reduce the ambiguity in the annotation process and ensures the selection of the most reliable annotation. However, if annotations of the same node differ in their predicted molecular formula, then APEX keeps both annotations. Although the APEX workflow focuses mainly on the annotation of first neighbors, it can also annotate those second neighbors (i.e., the nodes that are at a distance equal to two from the seed nodes, where the distance is equivalent to the minimum number of edges in the path between any pair of nodes) that are connected in all of the experimental networks.

### Application and Validation of the APEX Workflow to Identify GPNAE

**Figure 3:**
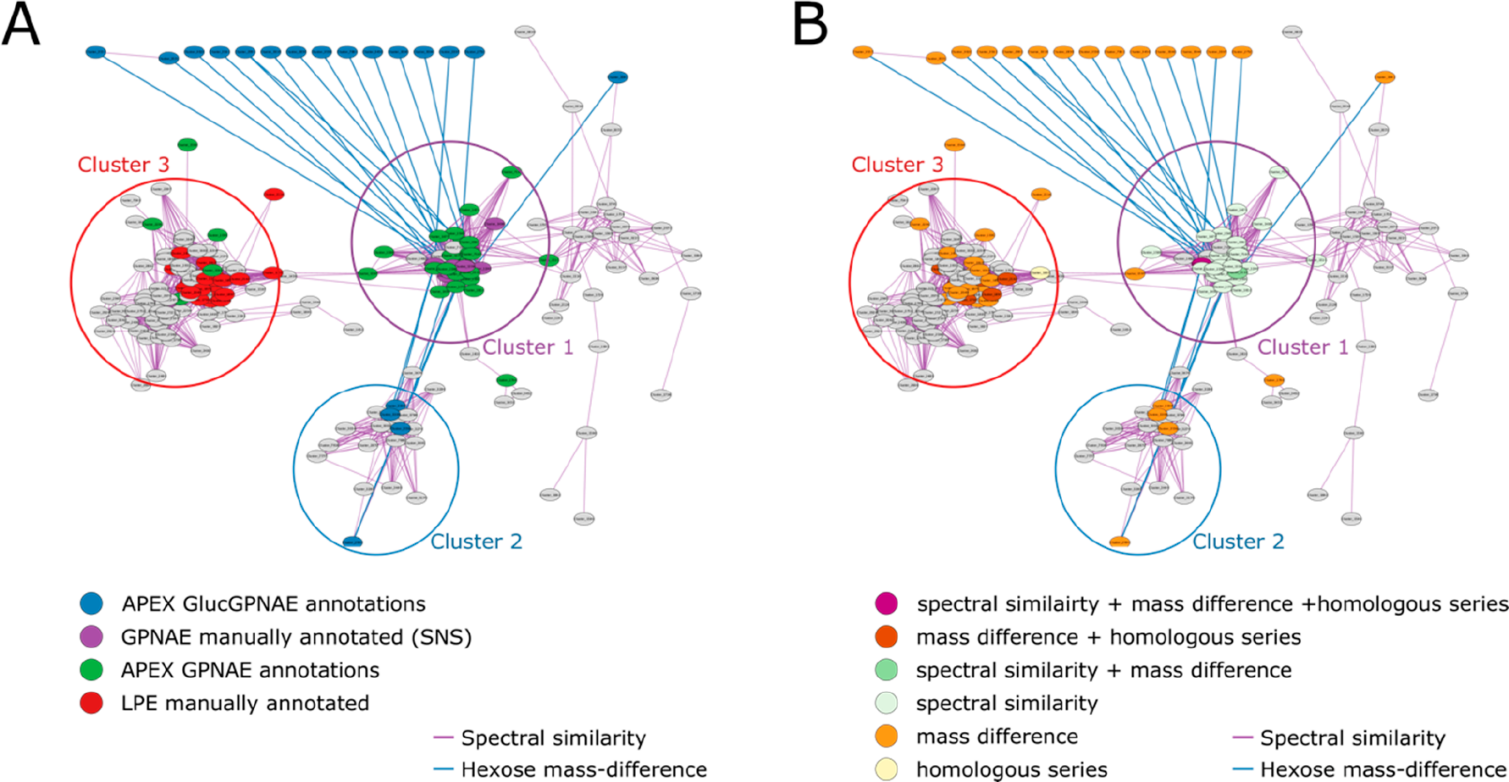
Spectral similarity network (threshold > 0.8) of data set 1 and APEX results using 10 manual annotations as seed node set (SNS). Spectral similarity edges are visualized in purple, and hexose mass-difference (162.0528) is in blue. There are different clusters formed in the network representing GPNAEs (cluster 1), GlycoGPNAEs (cluster 2), and Lysophosphatidylethanolamines (LPEs; cluster 3) (A) node coloring: purple: manual annotations/SNS; blue: APEX GlycoGPNAE annotations; green: remaining APEX-based annotations; red: manually annotated LPEs (B) APEX annotation levels, i.e., combination of edges; node coloring: purple: spectral similarity + mass difference + homologous series; red: mass difference + homologous series; green: spectral similarity + mass difference; light green: spectral similarity; orange: mass difference; yellow: homologous series.

We first tested our developed APEX workflow to annotate GPNAE in our in-house data set. In total, we manually annotated 19 GPNAE of different chain lengths and degrees of saturation that are all part of the same cluster in the spectral similarity network (cluster 1 in Figure 3A). We used all 19 manually annotated nodes as Seed Node Set (SNS) for the APEX workflow. As a result, we were able to annotate most nodes from cluster 1, mainly using the spectral similarity network in combination with the mass difference network, as shown in Figure 3B. All annotations, manually and predicted by APEX, reveal that cluster 1 corresponds to (unmodified) GPNAE varying in their number of alkyl chains and saturation.

The APEX workflow was able to connect the GPNAEs (cluster 1) to the separated GlycoGPNAEs (cluster 2) using the mass difference network; with the mass difference of 162.0528 (blue edges), corresponding to a hexose moiety. Figure 3 exclusively visualizes the mass differences of hexose molecules, highlighting the interconnectedness facilitated by APEX between the clusters and enabling their annotation (Figure 3B).

Even more, 12 GlycoGPNAE were separated from cluster 2 (in the merged network) and did not even appear in the spectral similarity network. This is because our in-house data set showed low MS^2^ coverage (30% in negative mode). As a result, most of the features are not present in the spectral similarity network, but since mass difference networks rely on MS^1^ data, those features can be also addressed and annotated using the APEX workflow.

Table 1 shows most of the APEX-based annotations of our in-house *C. elegans* microbiota data set are based on mass differences (specifically, 142 consider mass difference edges between nodes that are not connected via spectral similarity). But as mentioned, mass differences are less reliable than spectral similarity because connections to random features without biological meaning might arise. Even more, mass difference networks do not distinguish different potentially present isomers with different retention times, and as a result, they are also connected. GPNAEs are isomeric to Lysophosphatidylethanolamines (LPEs). Therefore, using only mass differences led to erroneous annotation of 23 LPEs that were annotated as GPNAEs by the APEX workflow, as shown in Figure 2. But since they had associated fragmentation spectra which showed a different fragmentation, corresponding nodes exist in the spectral similarity network and they are disconnected, differentiations can be made. Furthermore, in most cases isomeric GPNAEs and LPEs could be baseline separated in the chromatographic dimension.

**Table 1:** Overview on the Number of APEX Annotations through the Different Datasets and Metabolite Classes

	data set 1 in-house	data set 2 massive MSV000087885	data set 3 massive MSV000086293	
class	GPNAE	GPNAE	ascr	MOGL
number of seed nodes	19	10	9	16
total annotations	204	293	75	226
mass difference + spectral similarity + homol. series	14	11	0	0
mass difference + spectral similarity	10	11	10	23
mass difference + homol. series	23	46	1	0
spectral similarity + homol. series	2	0	0	0
spectral similarity	8	22	7	135
mass difference	142	192	57	68
homol. series	5	11	0	0
multiple annotations	114	241	32	155
multiple conflicting annotations	3	0	0	0

In order to further evaluate the APEX workflow, we reprocessed the publicly available data set from Helf et al.,^12^ which also detected GPNAEs alongside their glycovariants, and similar to the clustering of our in-house *C. elegans* microbiota data set, GPNAEs and GlycoGPNAEs were found in different, isolated clusters in the spectral similarity network. As we mentioned, this is due to the structural difference caused by the glucose moiety, leading to different fragmentation (see above).

We manually annotated 10 GPNAEs (using MS^1^ and MS^2^ data) that were used as SNS to annotate their neighbors by APEX, which resulted in the annotation of 293 nodes (Table 1): The APEX workflow also annotated 26 glycoGPNAEs using connections in the mass difference network. Even more, 12 out of those 26 glycoGPNAE annotations were made without the availability of MS^2^ spectra, which highlights the strength of our approach in annotating metabolites in cases where fragmentation data are not available.

The inclusion of homologous series to filter the mass difference network allows for potentially filtering out isomeric features with mismatching retention times (Table 1). Moreover, it adds additional confidence for features that have been annotated only on the MS^1^ level.

Additionally, for the current implementation of the APEX workflow, it is crucial that all species of a homologous series are present in the analysis, which is due to limitations of the *nontarget* R package used for generating the homologous series. This can be an issue for low-abundance species that are not detected in MS analysis.

The ability to utilize multiple experimental networks is a key strength of the APEX workflow, which can potentially overcome the limitations of using a single network and increase the accuracy and confidence in metabolite annotation.

### Comparison of Molecular Formula Predictions: APEX vs SIRIUS

To benchmark the proposed APEX workflow, we performed a comparison of the molecular formulas predicted by our approach with those obtained from SIRIUS, a widely used software tool for metabolite annotation in LC-MS/MS-based metabolomics that can be used to predict molecular formulas using isotopic patterns and fragmentation trees.^20^ While SIRIUS primarily focuses on calculating the best fitting formulas, APEX goes beyond that by leveraging additional biochemical information to refine and enhance formulas propagation.

To ensure a comprehensive comparison, we considered all candidate molecular formulas provided by SIRIUS, i.e., those with multiple candidates ranked based on their similarity to the observed spectrum. However, because of potentially missing MS^2^ data, not all features in the data set have molecular formulas available in SIRIUS computations. It is important to note that the APEX workflow provides molecular formulas only for features that are connected in the mass difference network, propagating the formula difference. Despite these limitations, we observed matches at 90.9% (i.e., 76 out of 83) in all APEX-based annotations for features with available molecular formulas and SIRIUS formula results (SIRIUS results available at https://github.com/michaelwitting/APEX).

To further validate the results of the APEX workflow, we manually annotated 93 different compounds (beyond the 19 GPNAE used as SNS, i.e., organic acids, amino acids, fatty acids, nucleotides, and glycerophospholipids; ids available at GitHub) in our in-house *C. elegans* microbiota data set and compared the observed molecular features with those obtained using the APEX workflow. Remarkably, we found no mismatches between the manually annotated compounds and the APEX-based annotations, providing compelling evidence for the accuracy and reliability of the APEX workflow.

Among the APEX-based annotations from the publicly available data set (MassIVE MSV000087885), 88.1% of the predicted formulas (i.e., 37 out of 42) matched those from SIRIUS. Notably, all (i.e., 3 out of 3) of the annotations based on the combination of spectral similarity, mass difference, and homologous series have the same molecular formula as those predicted by SIRIUS, which underscores their high reliability.

Additionally, we assessed the performance of the APEX workflow using leave-one-out cross-validation on our in-house *C. elegans* microbiota data set. This involved utilizing all but one manual annotation as the SNS and keeping the left out manual annotation as a Validation Set (VS). This process is repeated for each of the manual annotations, resulting in *n* number of evaluations, where *n* is the number of manual annotations. We also validated the APEX workflow using leave-two-out cross-validation. In both cases (leaving one or two seeds out at a time), all the VS were correctly annotated (i.e., matching molecular formulas).

To further evaluate the influence of the number of seed nodes on the APEX workflow results, we applied the leave-one-out cross-validation on three different approaches using all (i.e., 19), 50% (i.e., 10), and 25% (i.e., 5), of the manual GPNAE annotations as SNS, respectively. The VS for each approach was the set of left-out annotation. Remarkably, for all three approaches, each left-out VS was annotated correctly. This suggests that the APEX workflow performs well and generates reliable annotations, even with a small SNS.

### Influence on Seed Nodes

**Figure 4:**
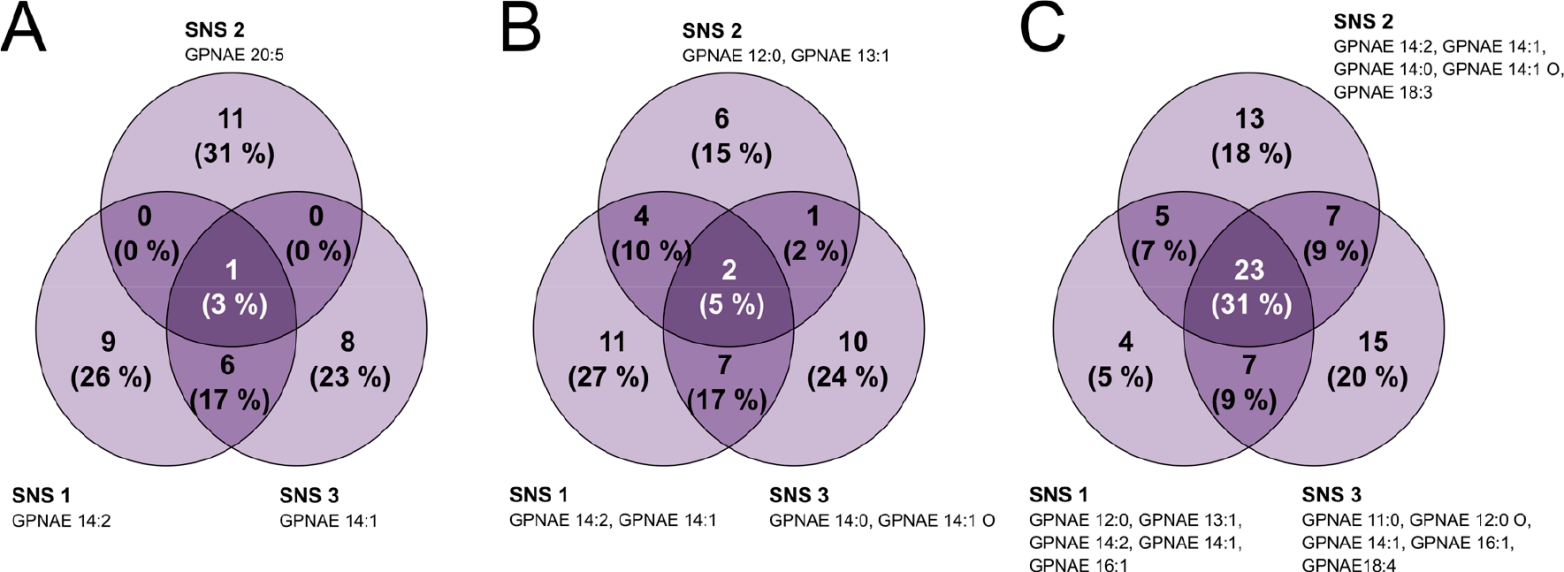
Consistency of annotations made by the APEX workflow using different (random) SNS of different sizes. (A) Overlap of annotations using three different (individual) nodes as SNS. (B) Overlap of annotations using three different pairs of nodes as SNS. (C) Overlap of annotations using three different SNS of five nodes each.

We evaluated the impact of the use of different Seed Node Sets (SNS) on the APEX workflow by randomly selecting three SNS of different sizes (1, 2, and 5 nodes) and assessing their influence on the number of observed APEX-based annotations. The SNS used determined the annotation process (Tables S3–S5). Our results showed that using different SNS resulted in relatively low overlap of the annotated features, especially when only one seed node was used (Figure 4A); whereas using more manual annotations as the SNS (Figure 4C) increased the overlap of APEX-based annotations between different SNS. We recommend using at least five manual annotations as the SNS to enhance annotation coverage of GPNAEs. The selection of seed nodes can significantly impact the annotation made by the APEX workflow, so researchers should carefully consider the number and combination of seed nodes used. To assess the overlap of true GPNAE annotations between different SNS, we performed a comparison by only retaining APEX-annotations that were annotated by the combination of spectral similarity, mass difference, and homologous series. We found that the overlap of these annotations increased with the size of the SNS (however, no trend was shown because the APEX-annotation strongly depends on the type of SNS). We compared the molecular formulas predicted by the APEX workflow with other manually annotated features of the data set and determined that using a minimum of 5 seed nodes as input yielded 36 matching formulas, ensuring high-quality annotations. In general, the larger the SNS, the more APEX-based annotations there will be, but the selection of seed nodes impacts the results. The use of multiple seed nodes is recommended to increase the quality and certainty of the APEX-based annotations.

### Validation on an Independent, Publicly Available Data Set

Finally, we tested the APEX workflow on a different data set focusing on two other compound classes in *C. elegan*s: ascarosides and modular glucosides (MOGLs). Ascarosides are signaling compounds involved in a wide range of biological processes, including development, reproduction, and behavior.^21−24^ Similar to GPNAEs, no reference spectra exist in public MS^2^ databases and no reference standards are commercially available. MOGLs have been recently described in *C. elegans* and are constructed through various combinations of diverse metabolic building moieties,^25^ making them an ideal model for evaluating APEX.

We used the data set from Le et al. (MSV000086293), in which we manually annotated 9 ascarosides (also reported in the respective publication, i.e., 8x ascr# and 1x icas#) and 16 MOGLs (i.e., 4x tyglu#, 11x iglu#, and 2x angl#), that we used as SNS.^17^

Starting with the ascarosides as seed nodes, the APEX workflow annotated 7 species (i.e., nodes) using only spectral similarity networks, 10 by a combination of spectral similarity and mass difference and 57 based only on mass difference (Table 1). The annotations obtained solely from the mass difference analysis and those obtained through a combination of mass difference and spectral similarity analysis lead to the annotation of similar variants, i.e., CH_2_, C_2_H_4_, HPO_3_, H_2_, C_2_H_2_, and C_9_H_5_NO variants. These annotations arise from differences in molecular formulas between various compounds or between an ascr# and its corresponding icas# variant related to an indole carboxylic acid residue. Therefore, connections in the mass difference network allow one to annotate similar variants, even if the corresponding nodes are not connected in the spectral similarity network. This is different from GPNAEs, where we noticed that Hexose mass differences only occurred when there were no spectral similarity edges present. By using APEX, we were able to annotate additional ascarosides, such as bhas#9 by the mass difference 44.0262 (C_2_H_4_O) to the seed node ascr#5 that resulted in the chemical formula C_11_H_20_O_7_, or phascr#71 by connecting ascr#7 with the mass difference of 79.9663 (corresponding to HPO_3_).

The same data set also included MOGLs that we aimed to annotate by APEX using a different set of seed nodes and observed 226 annotations (Table 1; 135 via spectral similarity, 68 via mass difference, and 23 via combination of both). Interestingly, the main mass differences were C_5_H_6_O, C_6_H_10_O_5_ (Hexose), C_13_H_22_O_5_ (corresponding to an ascr#1 block), CH_2_, and HPO_3_. Additionally, it is worth noting that the proportion of matching molecular formulas, when compared to the formulas predicted by SIRIUS, is slightly higher for the combination of mass difference and spectral similarity (82.6%, i.e., 19 out of 23) than for the mass difference annotations alone (71.7%, i.e., 30 out of 42).

Using APEX, we found 10 species connected to angl#4 (7 through spectral similarity, 2 through mass difference, and 1 through spectral similarity combined with mass difference). A particular example was the annotation of angl#4 + C_5_H_6_O with the molecular formula C_25_H_29_N_2_O_12_P, which potentially corresponds to angl#26 and was not reported in a previous publication. Even more, we annotated one feature with the potential chemical formula C_22_H_30_NO_10_P by the mass difference of C_3_H_6_O to iglu#8, which potentially corresponds to phicas#11. Another feature was potentially annotated as anglas#2 by APEX and the mass difference of C_13_H_22_O_5_ (which corresponds to an ascr#1 unit) to angl#2.

In conclusion, the APEX workflow was successfully applied to annotate additional species of ascarosides and MOGLs in *C. elegans*. By using spectral similarity and incorporating mass difference, a total of 75, and 226 species (ascarosides and MOGLs, respectively) were annotated. However, since both ascarosides and MOGLs do only rarely form homologous series, the filtering step previously applied to GPNAEs which can be categorized to lipid and lipid-like compounds could not be used here. Therefore, while the APEX workflow is effective for identifying ascarosides and MOGLs, it has some limitations that must be considered when annotating these compounds.

## Conclusion

Here we introduced an APEX workflow and used it for the annotation of glycerophospho *N*-acyl ethanolamides (GPNAEs), a compound class in *C. elegans*. The combination of spectral similarity, mass-difference, and homologous series allowed for accurate and comprehensive annotation of GPNAEs, including automated annotation of Glyco variants that are not connected in the spectral similarity network. The incorporation of different network tools improved the accuracy and comprehensiveness of the annotation process, while the quality of annotations was underscored by their high matching rate with the SIRIUS results. Additionally, the homologous series was introduced to filter out non-biological features and improve the identification of compounds with more biological significance. However, it is still necessary to use spectral similarity as a more reliable network since mass differences tend to be noisier and GPNAEs are isomeric to lysophosphatidylethanolamines, leading to incorrect annotations using the APEX workflow.

Moreover, the selection of the manual annotations used as the Seed Nodes Set (SNS) significantly influences the resulting APEX annotations, and a higher number of seed nodes enhances the annotation process. These results demonstrate the usefulness of the APEX workflow in identifying and characterizing compounds in complex data sets, particularly for glycolipid-related compounds.

In the future, different possibilities for the extension of APEX exist. GSMNs capture knowledge on known metabolic pathways and transformations and potentially allows to bridge individual features or cluster using biochemical reactions.^26,27^ Another possibility is the use of correlation networks. Especially, Gaussian graph models have been shown to be able to reconstruct biochemical valid links from metabolomics data.^28^ Furthermore, it helped to in identifying novel metabolites.^29^ By optimizing the selection of SNS and incorporating different complementary networks such as GSMNs, correlation networks, etc., the APEX workflow may provide even more comprehensive annotation results and become an invaluable resource for researchers seeking to decipher the complexities of the metabolome.

## Data Availability

The APEX workflow, networks and ids are available on GitHub (https://github.com/michaelwitting/APEX).

## Supporting Information Available

Pseudocode of the APEX algorithm (PDF)
Transformation file used for GNPAE annotation, transformation file used for ascaroside and MOGL annotation, overlap of molecular formulas predicted by APEX, overlap of molecular formulas predicted by APEX and manually annotated compounds, number of APEX annotations depending on number of seed nodes (XLSX)
