# Supplementary material for: APEX: an Annotation Propagation Workflow through Multiple Experimental Networks to Improve the Annotation of New Metabolite Classes in Caenorhabditis elegans
Source: Anal Chem. 2023 Nov 20;95(48):17550–8. doi: 10.1021/acs.analchem.3c02797 (PMC10701709; doi:10.1021/acs.analchem.3c02797)
Supplement: Supplementary file 1 — ac3c02797_si_001.pdf [file ac3c02797_si_001.pdf]

Supporting Information

APEX – an annotation propagation workflow through multiple experimental networks to improve the annotation of new metabolite classes in *Caenorhabditis elegans*

Liesa Salzer[1], Elva María Novoa-del-Toro[2], Clément Frainay[2], Kohar Annie B. Kissoyan[3], Fabien Jourdan[2,4], Katja Dierking[3], Michael Witting[5,6*]

[1] Research Unit Analytical BioGeoChemistry, Helmholtz Zentrum München, 85764 Neuherberg, Germany

[2] Toxalim (Research Centre in Food Toxicology), Université de Toulouse, INRAE, ENVT, INP-Purpan, UPS, 180 chemin de Tournefeuille St-Martin-du-Touch, BP 3, 31931 Toulouse Cedex, France

[3] Department of Evolutionary Ecology and Genetics, Zoological Institute, Kiel University, 24118 Kiel, Germany

[4] MetaToul-MetaboHUB, National Infrastructure of Metabolomics and Fluxomics, 180 chemin de Tournefeuille St-Martin-du-Touch, BP 3, 31931 Toulouse Cedex, France

[5] Metabolomics and Proteomics Core, Helmholtz Zentrum München, 85764 Neuherberg, Germany

[6] Chair of Analytical Food Chemistry, TUM School of Life Sciences, Technical University of Munich, 85354 Freising-Weihenstephan, Germany

* corresponding author:

Dr. Michael Witting, michael.witting@helmholtz-munich.de

S1 Overview of the APEX workflow

The automated **A**nnotation **P**ropagation through multiple **EX**perimental networks (APEX) workflow was defined as follows:

```
for each manually annotated seed node S_i (GPNAE / ascarosides):
   get the set of neighbors N(S_i)

      for each neighbor node N_j in N(S_i):
          for each mass difference edge connecting S_i to N_j:
              if m(N_j) < m(S_i) and the corresponding chemical formula (formula(S_i)
              - formula(N_j)) is not in the chemical formula of S_i:
                     remove the edge

              if N_j is not annotated yet:
                     annotate N_j with the chemical formula corresponding to
                     (formula(N_j) +/- formula(S_i)), if available

                     add new attributes to N_j containing the networks connecting N_j
                     and S_i, the seed node S_i, and other available attributes (i.e.
                     values of spectral similarity, mass-difference, and homologous
                     series)
              else:
                     if the number of networks connecting N_j and S_i is greater than
                     the current annotation or if the number is the same but the mass
                     difference |m(N_j) - m(S_i)| is smaller than the existing
                     annotation:
                            replace the existing annotation with the new one by:

                            annotate N_j with the chemical formula corresponding to
                            (formula(N_j) +/- formula(S_i)), if available

                            add new attributes to N_j containing the networks
                            connecting N_j and S_i, the seed node S_i, and other
                            available attributes (i.e. values of spectral similarity,
                            mass-difference, and homologous series)
```
